# Editorial

**Published:** 1901-01-15

**Authors:** 


### EDITORIAL.

WITH this number the DENTAL REGISTER enters upon its fifty-fifth year. Its past is a matter of history. That it has had an honorable as well as a prolonged existence is largely due to the fact that it has had the care and support of men who were eminent in the profession and filled with self-sacrificing devotion to its interests. It was not intended or expected that the present editorial management would be extended beyond the year 1900. Had Dr. Menges lived the DENTAL REGISTER would this year have entered upon a new and what promised to be the most important period of its existence. He was maturing plans for bringing to its support the pens of the best writers and workers in the dental world. By his death the REGISTER'S constituents and dental literature have experienced a loss which is to be greatly deplored.

The REGISTER has now passed into the possession and control of its present editor and publishers, and will be conducted in the future, as in the past, in the interest of the profession. While we claim the privilege of including within our province the whole dental world, we realize that our literature is ever expanding with the development of the profession and our limitations are necessarily somewhat restricted. We shall be content to serve a smaller constituency and give voice and record to its activities. We hope to be able to report as much as possible of the literary work done in the near limits of the center of population of the United States, which the recent census has located in Indiana, not far from the Ohio and Kentucky boundaries. We prefer not to locate definitely the outer limits of our claim for fear that we may encroach upon the preserves of other worthy and deserving contemporaries. But we wish most cordially to extend the use of our pages to all dental societies as well as individuals tributary to the territory we naturally represent. We shall strive to deserve the confidence and esteem of our constituents not only by giving them an adequate representation, but we hope also to keep them in touch, so far as may be practicable, with professional advances throughout the world. Our experience of the past year has been pleasant and we trust not without reasonable benefit to all concerned. With more experience and cordial support may not the coming year be one of still larger usefulness!

---

THE eighth annual meeting of the *Institute of Dental Pedagogics* was held at Nashville, Tenn., December 27th, 28th and 29th. It was largely attended, over twenty dental colleges being officially represented, some of the schools from far distant places being represented by three and four delegates. The papers and discussions were of a high order and full of interest; in fact, there was a spirit of enthusiasm in the proceedings that characterizes this organization and claims the interest of those in attendance as does no other meeting of similar kind. This was the only dental meeting that we ever attended that all remained until the program had been completed. Three full days were consumed in discussing only methods of imparting instruction. It is not too much to say that every one of the nearly seventy-five teachers present went home not only with some new ideas of teaching, but with a new conception of the high and noble calling in which he is engaged, and an awakened spirit which will take from his class work the burden of care which too often makes teaching perfunctory and ineffective. Nashville is the great educational center of the South, and this, combined with the proverbial Southern hospitality, made the occasion one of delight as well as profit.

---

THE annual clinic of the Chicago Dental College alumni, which has become famous because of the large numbers in attendance, and valuable new clinical methods and devices which are annually brought to notice through this organization, will be held at the Chicago Dental College, January 23d. It promises to be as largely attended and profitable as ever this year. Invitations are cordially extended to all practitioners to attend and take part in the clinics. Particulars may be had by addressing Dr. Hart J. Goslee, 580 W. Madison Street, Chicago.

---

WE greatly regret that our esteemed contemporary, the *Indiana Dental Journal*, has ceased publication. To say that it was unique expresses only certain characteristics which were perhaps most apparent. It was more than this, it was vigorously professional. Its contributions of original matter were many and of a high order and its selected matter showed discriminating taste, and we are sorry that it was not financially supported so that Brother Hunt should not have been compelled to give way to that disgusting "tired feeling." We shall miss this journal from our exchanges, but we trust that so wise and witty a pen may not long lie idle. We quote below the notice of suspension:

REST IN PEACE!

We announce the suspension of publication of the *Indiana Dental Journal* with the close of Volume III. We do not announce it with great regret, for if regret had been poignant, suspension of publication would not have occurred. Nor do we make it known with unmixed joy, for a measure of regret tinctures the announcement.

Further publication is suspended because the editor is tired. For the past twelve months he has been publisher, as well as all kinds of editor, and this, added to numerous other duties, made the situation no joke. The *Indiana Dental Journal* was first issued in January, 1898. It was conceived, started, paid for, conducted and edited by two dentists for the first two years. No other persons, no firm, corporation, association, municipality or government ever had any interest, real or otherwise, in its publication. It was designed to foster the best interests of the profession in Indiana, and to incidentally adjust any loose joints in the dental mechanism of the whole country. The first object was attained, to a degree, the second, only partially achieved. There are still a few questions left unsettled, and still a few editors with whom we have not exchanged editorial amenities. But you can not have everything in this world.

Our editorial experience of the past three years has been, in many ways, a pleasant one; in every way an interesting one. We have nothing to "take back," nor, on the whole, do we see many neglected opportunities in the past thirty-six months. In fact, we are quite well satisfied with the record. Starting with absolutely no blare of trumpets, and without a subscriber, we put out a journal that gradually extended its sphere of influence until it was being read in forty-four States—from Maine to Washington and from Minnesota to Florida, and in Manila, England, Canada and Cuba, by bona fide subscribers. That was worth the doing, was it not?

But the grind of it! Some of those thirty-six numbers were put together on the train, editorials were written in various hotels at numerous places. Sundays, that should have been devoted to pious meditation, were sacrificed to editorial work, and evenings that might have been profitably used for sandpapering the intellect were immolated on the altar of original contributions. These are the things that we do not regret.

To all those who advertised with us and thus contributed to making the *Journal* a possibility, we extend our thanks. To those who so loyally stood by us and sent in their $1.00 per annum, we grovel. The numerous kindly letters of these latter were the chief solace of our editorial life. To part with you is our regret.

THE INDIANA DENTAL JOURNAL,
BORN JANUARY, 1898,
DIED DECEMBER, 1900.
NO COMPLAINT, EVERYBODY SATISFIED.
CAUSE OF DEATH, THE EDITOR WAS TIRED.

THE prospectus of the new volume of the *International Dental Journal* is before us, together with an appeal from the board of managers for the continued support of the profession. We regard the *International Dental Journal* as one of the ablest dental publications. It well deserves the support of the profession, not so much because it is an independent journal and dependent wholly on the profession for its support, but because it is so ably conducted. Its editorials are timely and characterized by a dignified and fearless comment on current events, which alone ought to place this journal in the hands of every progressive dentist in the land. Its contributed papers have always been of a high order.

THE *Dental Brief* starts the new century in a new dress. It is larger in form and printed with new and beautiful type on excellent paper. The cover is dark gray with red and blue printing and is not very pretty. We are sorry the size of the page has been changed as it makes an odd size when bound. But we shall overlook these trifles if the editor's New Year's greeting be realized in the new volume.

The Foreign Relations Committee, of the National Association of Dental Faculties, seems to be having some difficulty in convincing the executive officers of some of our dental colleges that it has any right to interfere with the reception of foreign students with improper credentials. The committee may not be so empowered, but it is doing a very proper work in calling attention to the loose methods used in passing on foreign credits which have heretofore obtained. We trust that very soon some well defined rules of conduct shall be laid down by the Faculties Association. We notice in a recent daily newspaper that a man practicing dentistry in Berlin, Germany, with a bogus American Degree had been arrested and fined and compelled to quit practicing, all because of the work of this committee.

We regret to learn of the death of Col. John E. Robie, the treasurer of the Buffalo Dental Manufacturing Company. He will be greatly missed by a large circle of friends and by his associates in business. As a business man he was courteous and successful, and much of his firm's prosperity is due to his untiring zeal and enterprise. He was not old, being in his fifty-fifth year, but still he has done much to facilitate the practical work of our profession, for which we shall ever give him an honorable place in professional history.

The following are the officers elected at the last meeting of the Ohio State Dental Society: President, H. F. Harvey, Cleveland; 1st Vice President, Otto Arnold, Columbus; 2nd Vice President, J. B. Bauman, Columbus; Secretary, S. D. Ruggles, Portsmouth; Treasurer, C. I. Keely, Hamilton.

